# Cognitive markers of preclinical and prodromal Alzheimer's disease in Down syndrome

**DOI:** 10.1016/j.jalz.2018.08.009

**Published:** 2019-02

**Authors:** Carla M. Startin, Sarah Hamburg, Rosalyn Hithersay, Tamara Al-Janabi, Kin Y. Mok, John Hardy, LonDownS Consortium, Andre Strydom, Elizabeth Fisher, Dean Nizetic, John Hardy, Victor Tybulewicz, Annette Karmiloff-Smith, Nidhi Aggarwal, Amy Davies, Lucy Fodor-Wynne, Bryony Lowe, Erin Rodger, Laura Checkley, Paul Gallagher, Bernice Knight, Anne-Marije Prins, Rory Sheehan, David Zhang, Andre Strydom

**Affiliations:** a Department of Forensic and Neurodevelopmental Sciences, Institute of Psychiatry, Psychology and Neuroscience, King's College London, London, UK; b Division of Psychiatry, University College London, London, UK; c The LonDownS Consortium, London, UK; d Department of Molecular Neuroscience, Institute of Neurology, University College London, London, UK; e Division of Life Science, Hong Kong University of Science and Technology, Hong Kong SAR, People's Republic of China; f Reta Lila Weston Institute, Institute of Neurology, University College London, London, UK

**Keywords:** Down syndrome, Alzheimer's disease, Dementia, Cognitive decline, Prodromal, Preclinical, Randomized controlled trials

## Abstract

**Introduction:**

Down syndrome (DS) is associated with an almost universal development of Alzheimer's disease. Individuals with DS are therefore an important population for randomized controlled trials to prevent or delay cognitive decline, though it is essential to understand the time course of early cognitive changes.

**Methods:**

We conducted the largest cognitive study to date with 312 adults with DS to assess age-related and Alzheimer's disease–related cognitive changes during progression from preclinical to prodromal dementia, and prodromal to clinical dementia.

**Results:**

Changes in memory and attention measures were most sensitive to early decline. Resulting sample size calculations for randomized controlled trials to detect significant treatment effects to delay decline were modest.

**Discussion:**

Our findings address uncertainties around the development of randomized controlled trials to delay cognitive decline in DS. Such trials are essential to reduce the high burden of dementia in people with DS and could serve as proof-of-principle trials for some drug targets.

## Highlights

• Cognitive changes with aging in DS are likely due to the progression of AD.
• Memory and attention show the earliest cognitive decline, from the early 40s.
• Memory abilities show the greatest sensitivity to dementia development.
• Modest sample sizes are sufficient for RCTs to prevent/delay dementia in DS.

## 1 Background

Down syndrome (DS), caused by trisomy of chromosome 21, has a UK incidence of approximately one in 1000 live births [1] and is associated with intellectual disability (ID) and an ultra-high risk of developing Alzheimer's disease (AD) [2]. The cumulative incidence of dementia has been suggested to be 95.7% by the age of 68 years with a mean age of diagnosis of 55 years [3], indicating cognitive decline is a near universal part of aging in DS. This increased dementia risk is driven by the overexpression of genes on chromosome 21, in particular the amyloid precursor protein (*APP*) gene; deposits of its protein product, amyloid-β, are a characteristic feature of AD and are found in the brains of adults with full trisomy 21 by the mid-30s [2], [4]. DS may therefore be viewed as a genetic cause of AD alongside known autosomal dominant pathogenic mutations in the *APP*, presenilin-1 (*PSEN1*), and presenilin-2 (*PSEN2*) genes (autosomal dominant AD [ADAD]) [5], although the clinical course during the early stages of AD in DS is less well described [6].

Understanding the time course of AD in DS is essential to improving detection and monitoring of decline and will aid in designing intervention studies [7]. Given their AD burden, individuals with DS are an important population for clinical trials of treatments to prevent or modify AD, particularly for drugs targeting amyloid overproduction or deposition. We therefore aimed to understand neuropsychological changes across the time course of AD development at a population level in adults with DS using data from a large, ongoing, in-depth phenotyping study of the development of AD in DS [8]. We aimed to estimate the effect of the apolipoprotein E (*APOE*) ε4 allele, the best known genetic risk factor for AD aside from variations in *APP*, *PSEN1*, and *PSEN2*, on performance for outcomes most sensitive to cognitive changes. Using such outcomes as hypothetical clinical trial primary outcomes, we also aimed to estimate sample sizes for randomized controlled trials (RCTs) to delay cognitive decline either 10–15 years before the mean age of dementia diagnosis or alternatively around the mean age of diagnosis.

## 2 Methods

### 2.1 Participants

We recruited 312 individuals with a clinical diagnosis of DS aged 16+ years across England and Wales. Full details regarding participants and the assessment can be found in the study by Startin et al. [8].

Ethical approval was obtained from the North West Wales Research Ethics Committee (13/WA/0194). Where individuals had capacity to consent for themselves, we obtained written informed consent. Where individuals did not have capacity to consent, a consultee was asked to approve the individual's inclusion based on their knowledge of the individual and his/her wishes, in accordance with the UK Mental Capacity Act 2005.

### 2.2 Genetic analysis

DS was confirmed genetically in 299 individuals using saliva or blood samples. The *APOE* genotype was determined using a Thermo Fisher Scientific TaqMan assay for SNPs rs7412 and rs429358 (Waltham, MA).

### 2.3 Assessment

Our assessment battery (Table 1) included cognitive tests completed with individuals who were able to engage in assessment and who met vision and hearing screening thresholds, and informant ratings from relatives or paid carers who knew the individual well for all participants [8]. Informant measures are important for adults who cannot engage in cognitive assessments, who have vision or hearing difficulties, or who are at floor on cognitive tests. Our battery has previously been validated and adapted for use in older adults with DS, including those with little verbal ability [8], [23], and focuses on abilities related to memory, executive function, and motor coordination, as these are often impaired in DS [19] and further impaired by dementia [23].

**Table 1:** Summary of assessments used

Domain	Test name	Primary ability assessed	Description	Outcomes and score ranges
IQ	Kaufmann Brief Intelligence Test 2 (KBIT-2) [9]	General cognitive abilities	Subtests assess participants' verbal abilities (verbal knowledge and riddles) and nonverbal abilities (matrices).	Verbal raw score (0–108); Nonverbal raw score (0–46)
Memory	CANTAB paired associates learning (PAL) [10]	Visuospatial associate memory	Participants were required to remember locations of an increasing number of patterns hidden behind boxes on a computer screen.	First trial memory score (0–26); Number of stages completed (0–8)
	CAMCOG orientation [11]	Orientation	Assesses participants' knowledge of when it is and where they are.	Total score (0–12)
	Object memory test [12]	Recall memory	Participants were required to name and remember a series of objects, then recall them in two immediate trials and one 5-minute delayed trial.	Immediate recall (0–14); Delayed recall (0–7)
	Dementia Questionnaire for People with Learning Disabilities (DLD) [13]	Memory and orientation	Informants answer 22 questions about abilities associated with cognitive decline over the last 2 months.	Cognitive abilities (0–44)
	Observer Memory Questionnaire (OMQ) [14]	Memory	Informants answer 30 questions about individuals' memory abilities over the last 2 months.	Total score (30–150)
Executive function	CANTAB intra-/extra-dimensional set shift (IED) [10]	Rule learning and set shifting	Participants were required to learn rules about which was the “correct” of two presented patterns on a computer screen, with a rule change after 6 consecutive correct trials.	Number of stage 1 errors (0–50); Number of stages completed (0–9)
	Verbal fluency [15]	Verbal fluency	Participants were asked to name as many animals as possible in 1 minute.	Number of unique animals (0–N/A)
	Tower of London [16], [17]	Working memory and planning	Participants were required to move beads on a board to match presented configurations.	Total score (0–10)
	Behavior Rating Inventory of Executive Function–Adult version (BRIEF-A) [18]	Executive function	Informants answer 70 questions about problems with behaviors relating to executive functioning over the last month.	Total score (70–210); Behavioral Regulation Index (30–90); Metacognition Index (40–120)
Attention	CANTAB simple reaction time (SRT) [10], [19]	Attention/motor abilities	Participants were required to press a button as soon as a white square appeared on a computer screen.	Total number of correct responses (0–100); Mean latency (N/A); Latency standard deviation (N/A)
Motor	Finger-nose pointing [20]	Motor coordination	Participants alternatively touch their nose and a red circle 45 cm away for 20 seconds.	Total number of times the circle is touched (0–N/A)
	Developmental NEuroPSYchological Assessment-II (NEPSY-II) visuomotor precision [21]	Hand-eye coordination	Participants were timed as they traced around train, car, and motorbike tracks, with time and number of errors for each track used to determine overall scores.	Train and car score (0–30); Car and motorbike score (0–52)
Adaptive	Short Adaptive Behavior Scale (short ABS) [22]	Adaptive abilities	Informants answer 24 questions about everyday adaptive abilities.	Total score (0–113); Personal self-sufficiency (0–33); Community self-sufficiency (0–48); Personal-social responsibility (0–32)
	Dementia Questionnaire for People with Learning Disabilities (DLD) [13]	Adaptive abilities	Informants answer 28 questions about behaviors associated with cognitive decline over the last 2 months.	Social abilities (0–60)

#### 2.3.1 Missing data

Some adults, in particular those with dementia, had difficulty engaging in cognitive tests [8]. Excluding such adults could bias analyses. We therefore imputed scores (see Supplementary Table 1; total 14.1% of scores) as follows: individuals who were clearly unable to understand task instructions were allocated a score of zero where appropriate and when not appropriate were allocated a score to indicate poor performance. For *IED stage 1 errors*, a score of 25 was given (representing performance by chance). For *SRT total correct*, the minimum obtained score in our sample was given, whereas for *SRT mean latency* and *SRT latency standard deviation*, the maximum obtained scores in our sample were given (representing the poorest performance observed); these values were 13, 2500.61 ms, and 980.98 ms, respectively. Finally, when the *KBIT-2 riddles* subtest was incomplete, this score was imputed based on the linear relationship between the *riddles* and *verbal knowledge* subtest scores in our sample (*r* = 0.869, *P* < .001), and the *KBIT-2 verbal raw score* was calculated using this imputed score.

Missing items from the Dementia Questionnaire for People with Learning Disabilities (DLD), Observer Memory Questionnaire (OMQ), and Behavior Rating Inventory of Executive Function–Adult version (BRIEF-A) were imputed for up to 15% of items within each domain with the nearest integer to the mean value of completed scores. Questionnaire domains containing more than 15% of missing items were excluded from analyses.

### 2.4 Statistical analysis

SPSS, version 22, was used for analyses. Age and demographic factors were compared between groups using two-sample *t*-tests and χ^2^ tests, respectively. To account for multiple comparisons, *P* < .01 was used to determine statistical significance.

#### 2.4.1 Earliest cognitive markers of AD-related neuropathology

Because virtually all individuals with DS develop AD neuropathology as they age, we hypothesized that age-associated differences in outcome measures would be related to the progression of AD pathology, and this effect can be used to identify the earliest markers of cognitive decline. Based on the presence of amyloid neuropathology by the mid-30s [2], [4], performance of participants aged 16-30 years therefore represents abilities before the development of significant AD neuropathology and subsequent cognitive decline. We compared individuals' performance regardless of dementia status in 5-year age bands (31–35, 36–40, 41–45, 46–50, 51–55, and 56–60 years) against those aged 16–30 years using ANCOVAs, with premorbid ID severity and a measure of multimorbidity (presence of two or more common health conditions [24] excluding dementia and epilepsy developed after the age of 35 years) included as covariates to adjust for potential confounding effects. η^2^ values determined the overall effect size of age group. Pairwise comparisons with Bonferroni corrections determined age groups for whom performance was significantly poorer than that of those aged 16–30 years.

#### 2.4.2 Markers associated with clinical stage of AD

Preclinical (asymptomatic) AD can be defined as the stage when biomarker changes are present, but clinical symptoms have not yet developed, whereas prodromal AD is usually defined as the earliest symptomatic stage when cognitive symptoms are present, but the threshold for dementia diagnosis has not yet been reached [25]. Owing to postmortem studies indicating amyloid neuropathology in DS by the mid-30s [2], [4], we considered those aged 36+ years with no clinical symptoms of dementia to be in a preclinical state, and those with cognitive symptoms but no clinical diagnosis of dementia in a prodromal state. For participants aged 36+ years with no clinical dementia diagnosis, two ID psychiatrists independently reviewed detailed information on dementia symptoms using the Cambridge Examination of Mental Disorders of Older People with Down's syndrome and others with Intellectual Disabilities (CAMDEX-DS) [26] with diagnostic rating procedures described previously [27]. A consensus decision was made to allocate those with cognitive symptoms associated with AD but no evidence of decline in functional abilities and no other significant cause of decline to a prodromal dementia group, and asymptomatic individuals to a preclinical group. We then compared performance for adults aged 36+ years in a preclinical state to those in a prodromal state, and that of those in a prodromal state to those with a clinical diagnosis of dementia using ANCOVAs to identify markers of AD progression while controlling for age, premorbid ID severity, and multimorbidity, with η^2^ values to estimate the effect size of group.

#### 2.4.3 Sensitivity of cognitive markers to APOE genotype

To determine the effect of an *APOE* ε4 allele on performance for selected outcomes that were most sensitive to cognitive changes in adults aged 36+ years, we compared performance for those with genotype *APOE* ε3/ε3 and *APOE* ε3/ε4 using ANCOVAs while controlling for age, premorbid ID severity, and multimorbidity, with values of η^2^ to estimate the effect size of genotype.

#### 2.4.4 Sample sizes for RCTs using cognitive markers

We estimated sample sizes for two hypothetical disease-modifying RCTs using our participant sample and potential primary outcome measures, with the aim to delay individuals' abilities declining. Both trials were hypothesized to last 5 years, with the aim to prevent performance of adults aged 36–40 years declining to that of adults aged 41–45 years (i.e., delaying early decline) or to prevent performance of adults aged 46–50 years declining to that of adults aged 51–55 years (i.e., delaying later decline). Mean scores for the two relevant age groups were used to determine expected group differences (excluding individuals with clinical dementia in the younger age group as these individuals would be ineligible for such a trial), and expected group differences used with the associated pooled standard deviation (SD) to estimate the potential sample sizes needed for RCTs with *P* < .05 and 90% power. Owing to small sample sizes resulting in large confidence intervals for mean group differences, sample sizes were also estimated using the midpoint between mean group differences and their lower 95% confidence interval as a more cautious estimate for expected group differences. All sample size estimates were calculated by

$$n=2F{\left(\frac{\sigma }{d}\right)}^{2}$$

where *n* is the sample size needed per group, *F* is 10.51 (based on *P* < .05 and 90% power), *σ* is the pooled SD, and *d* is the expected group difference.

## 3 Results

### 3.1 Earliest cognitive markers of AD-related neuropathology

**Fig. 1:**
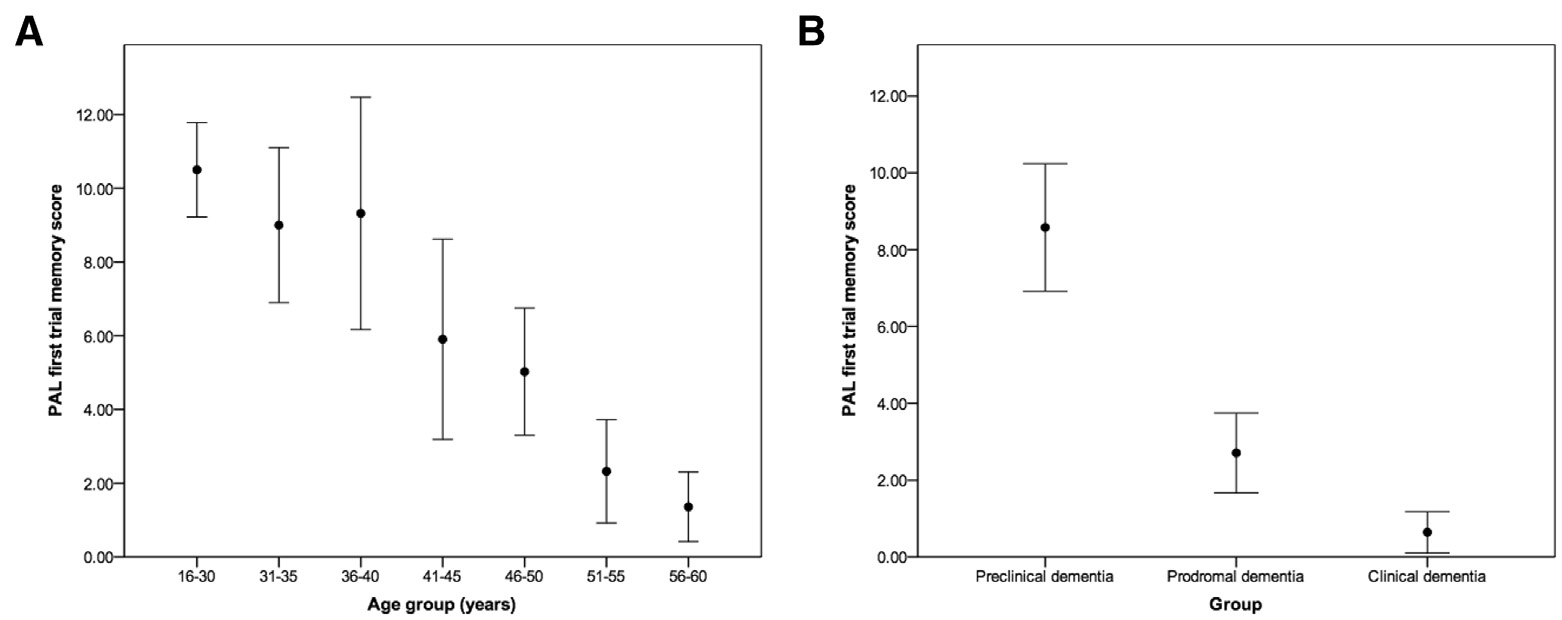
Mean PAL first trial memory scores for (A) different age groups and (B) preclinical, prodromal, and clinical dementia groups (error bars represent 95% confidence intervals).

Demographic information and scores for each outcome measure for 297 individuals aged 16-60 years split into age groups can be seen in Table 2; adults aged 61+ years (n = 15) were excluded due to small samples. All outcomes aside from BRIEF-A scores had a significant (*P* < .001) overall relationship with age group, with poorer performance in older age groups (see Fig. 1 for an example). Age group showed the greatest effect size as determined using η^2^ values for measures from the paired associates learning (PAL), object memory, SRT, and Developmental NEuroPSYchological Assessment-II explaining more than 30% of variance in scores for each outcome (Table 2).

**Table 2:** Demographic information and scores for each outcome for participants split by age group, with the overall effect of comparing all age groups

Demographics and outcomes	16–30 years	31–35 years	36–40 years	41–45 years	46–50 years	51–55 years	56–60 years	Age group comparison
Total number	94	30	27	24	52	42	28	N/A
Number failed hearing or vision test	2 (2.1%)	1 (3.3%)	2 (7.4%)	1 (4.2%)	2 (3.8%)	6 (14.3%)	1 (3.6%)	N/A
Age (years)	22.89 ± 4.11	32.60 ± 1.30	38.00 ± 1.44	43.17 ± 1.31	47.92 ± 1.31	52.83 ± 1.46	57.75 ± 1.40	N/A
Sex								
Male	46 (48.9%)	13 (43.3%)	14 (51.9%)	16 (66.7%)	30 (57.7%)	19 (45.2%)	11 (39.3%)	X(6) = 6.17, *P* = .404
Female	48 (51.1%)	17 (56.7%)	13 (48.1%)	8 (33.3%)	22 (42.3%)	23 (54.8%)	17 (60.7%)	
Premorbid ID severity∗								
Mild	33 (35.1%)	14 (46.7%)	10 (37.0%)	11 (45.8%)	26 (50.0%)	12 (28.6%)	8 (28.6%)	X(12) = 19.66, *P* = .074
Moderate	48 (51.1%)	16 (53.3%)	13 (48.1%)	9 (37.5%)	17 (32.7%)	17 (40.5%)	15 (53.6%)	
Severe	13 (13.8%)	0 (0.0%)	4 (14.8%)	4 (16.7%)	9 (17.3%)	13 (31.0%)	5 (17.9%)	
Ethnicity								
White	77 (81.9%)	24 (80.0%)	23 (85.2%)	21 (87.5%)	44 (84.6%)	38 (90.5%)	26 (92.9%)	X(6) = 3.79, *P* = .705
Nonwhite	17 (18.1%)	6 (20.0%)	4 (14.8%)	3 (12.5%)	8 (15.4%)	4 (9.5%)	2 (7.1%)	
Multimorbidity; number with two or more health conditions	63 (67.0%)	16 (53.3%)	17 (63.0%)	15 (62.5%)	32 (61.5%)	22 (52.4%)	13 (46.4%)	X(6) = 5.86, *P* = .439
Dementia status†								
Preclinical	N/A	N/A	16 (76.2%)	13 (56.5%)	25 (53.2%)	11 (29.7%)	1 (3.7%)	N/A
Prodromal	N/A	N/A	2 (9.5%)	8 (34.8%)	12 (25.5%)	10 (27.0%)	12 (44.4%)	
Clinical	N/A	N/A	3 (14.3%)	2 (8.7%)	10 (21.3%)	16 (43.2%)	14 (51.9%)	
Missing	N/A	N/A	6	1	5	5	1	
*APOE* genotype†								
ε2/ε2	0 (0.0%)	1 (3.4%)	1 (3.7%)	0 (0.0%)	1 (2.0%)	0 (0.0%)	1 (3.7%)	N/A
ε2/ε3	13 (14.3%)	6 (20.7%)	3 (11.1%)	3 (13.6%)	4 (8.0%)	3 (7.9%)	5 (18.5%)	
ε3/ε3	50 (54.9%)	16 (55.2%)	16 (59.3%)	12 (54.5%)	31 (62.0%)	25 (65.8%)	15 (55.5%)	
ε3/ε4	24 (26.4%)	6 (20.7%)	7 (25.9%)	7 (31.8%)	11 (22.0%)	9 (23.7%)	5 (18.5%)	
ε4/ε4	3 (3.3%)	0 (0.0%)	0 (0.0%)	0 (0.0%)	0 (0.0%)	1 (2.6%)	0 (0.0%)	
ε2/ε4	1 (1.1%)	0 (0.0%)	0 (0.0%)	0 (0.0%)	3 (6.0%)	0 (0.0%)	1 (3.7%)	
Missing	3	1	0	2	2	4	1	
KBIT-2 verbal raw score	33.76 ± 17.03	38.07 ± 16.32	32.92 ± 17.08	31.39 ± 18.74	24.56 ± 19.16	17.36 ± 16.33	10.19 ± 14.36	F(6,273) = 12.57, *P* < .001, η^2^ = 0.216
KBIT-2 nonverbal raw score	15.08 ± 7.41	14.69 ± 5.02	14.26 ± 7.90	12.32 ± 7.07	10.13 ± 7.07	8.54 ± 6.81	3.88 ± 5.57	F(6,265) = 14.69, *P* < .001, η^2^ = 0.250
PAL first trial memory score	10.50 ± 5.83	9.00 ± 5.31	9.32 ± 7.10	5.90 ± 5.97	5.02 ± 5.46	2.32 ± 3.82	1.36 ± 2.29	F(6,240) = 17.23, *P* < .001, η^2^ = 0.301
PAL stages completed	6.32 ± 2.68	5.96 ± 2.14	5.50 ± 3.17	4.57 ± 3.14	3.59 ± 2.92	2.16 ± 2.52	1.60 ± 2.25	F(6,240) = 19.21, *P* < .001, η^2^ = 0.324
Orientation	9.34 ± 3.77	10.24 ± 2.76	9.17 ± 4.03	7.62 ± 4.21	7.11 ± 4.77	5.69 ± 4.79	2.58 ± 3.50	F(6,257) = 14.36, *P* < .001, η^2^ = 0.251
Object memory immediate recall	10.16 ± 3.29	10.52 ± 1.92	8.77 ± 3.90	8.60 ± 4.11	7.83 ± 4.64	4.65 ± 4.47	2.32 ± 3.66	F(6,248) = 21.82, *P* < .001, η^2^ = 0.346
Object memory delayed recall	5.68 ± 1.65	6.10 ± 1.05	4.86 ± 2.17	4.50 ± 2.31	4.43 ± 2.60	2.62 ± 2.59	1.36 ± 2.00	F(6,248) = 22.42, *P* < .001, η^2^ = 0.352
DLD cognitive score‡	8.22 ± 8.68	5.50 ± 7.01	7.83 ± 9.50	13.52 ± 12.37	15.60 ± 12.81	21.52 ± 12.09	23.38 ± 10.54	F(6,252) = 16.09, *P* < .001, η^2^ = 0.277
OMQ total score‡	76.87 ± 18.15	68.17 ± 18.08	80.58 ± 22.51	81.21 ± 19.42	89.05 ± 23.87	95.41 ± 21.95	103.35 ± 18.02	F(6,244) = 10.25, *P* < .001, η^2^ = 0.201
IED stage 1 errors‡	4.18 ± 6.99	5.04 ± 8.70	6.64 ± 10.03	11.05 ± 11.57	10.71 ± 12.34	13.21 ± 10.64	20.21 ± 10.28	F(6,242) = 12.03, *P* < .001, η^2^ = 0.230
IED stages completed	6.52 ± 2.54	6.46 ± 2.83	6.05 ± 3.36	4.95 ± 3.38	4.64 ± 3.76	2.91 ± 3.34	1.08 ± 2.39	F(6,242) = 14.56, *P* < .001, η^2^ = 0.265
Verbal fluency	10.92 ± 6.08	10.52 ± 5.35	9.74 ± 6.59	8.14 ± 5.43	6.65 ± 6.25	4.54 ± 5.05	2.19 ± 3.66	F(6,260) = 12.01, *P* < .001, η^2^ = 0.217
Tower of London	7.22 ± 3.04	7.36 ± 3.34	7.00 ± 3.57	5.91 ± 3.68	4.57 ± 3.88	3.00 ± 3.66	2.35 ± 3.57	F(6,258) = 13.67, *P* < .001, η^2^ = 0.241
BRIEF-A total score‡	123.14 ± 25.59	115.04 ± 27.80	119.91 ± 23.04	122.53 ± 22.83	128.61 ± 29.25	131.11 ± 29.84	135.76 ± 30.16	F(6,230) = 1.47, *P* = .188, η^2^ = 0.037
BRIEF-A Behavioral Regulation Index‡	51.11 ± 11.81	49.55 ± 13.85	50.62 ± 10.77	50.85 ± 10.25	54.80 ± 12.84	52.45 ± 12.77	55.11 ± 12.73	F(6,250) = 1.13, *P* = .344, η^2^ = 0.026
BRIEF-A Metacognition Index‡	72.23 ± 16.70	66.46 ± 16.79	69.64 ± 14.97	72.16 ± 14.37	75.13 ± 18.25	78.36 ± 19.39	80.24 ± 18.11	F(6,231) = 1.58, *P* = .154, η^2^ = 0.039
SRT total correct	91.60 ± 16.20	93.67 ± 12.11	80.00 ± 31.11	81.47 ± 27.05	70.44 ± 34.31	52.06 ± 35.95	37.48 ± 33.85	F(6,232) = 20.54, *P* < .001, η^2^ = 0.347
SRT mean latency‡	692.50 ± 459.80	767.75 ± 526.72	1041.84 ± 778.43	1154.79 ± 553.89	1274.22 ± 764.30	1738.96 ± 763.32	2017.53 ± 713.45	F(6,232) = 21.84, *P* < .001, η^2^ = 0.361
SRT latency standard deviation‡	323.48 ± 229.30	317.83 ± 174.02	426.61 ± 296.51	536.88 ± 217.02	568.74 ± 295.18	733.28 ± 259.83	807.48 ± 244.49	F(6,232) = 23.42, *P* < .001, η^2^ = 0.377
Finger-nose pointing	10.99 ± 5.46	10.69 ± 4.70	8.83 ± 5.95	7.48 ± 5.11	6.59 ± 5.25	5.60 ± 5.93	2.73 ± 3.86	F(6,260) = 13.72, *P* < .001, η^2^ = 0.241
NEPSY-II train and car	15.83 ± 5.24	16.10 ± 5.43	15.00 ± 5.48	14.43 ± 6.45	8.98 ± 7.65	8.68 ± 7.94	4.77 ± 6.48	F(6,259) = 18.56, *P* < .001, η^2^ = 0.301
NEPSY-II car and motorbike	16.73 ± 9.32	17.83 ± 10.58	15.45 ± 9.05	12.14 ± 9.57	6.81 ± 8.49	6.06 ± 7.56	2.92 ± 4.77	F(6,259) = 17.89, *P* < .001, η^2^ = 0.293
Short ABS total score	76.72 ± 19.88	85.76 ± 17.68	76.75 ± 23.65	73.95 ± 26.21	68.13 ± 25.39	54.64 ± 27.56	50.17 ± 25.00	F(6,260) = 9.97, *P* < .001, η^2^ = 0.187
Short ABS personal self-sufficiency	28.63 ± 4.70	29.79 ± 3.90	27.84 ± 5.60	26.86 ± 5.12	25.89 ± 7.45	21.58 ± 9.40	20.42 ± 9.30	F(6,266) = 10.17, *P* < .001, η^2^ = 0.187
Short ABS community self-sufficiency	26.67 ± 10.28	31.24 ± 10.05	27.76 ± 11.51	25.70 ± 14.32	22.74 ± 12.23	17.64 ± 11.97	14.04 ± 10.44	F(6,264) = 9.08, *P* < .001, η^2^ = 0.171
Short ABS personal-social responsibility	21.78 ± 6.60	24.72 ± 5.61	20.96 ± 7.70	21.52 ± 7.96	19.70 ± 7.70	16.32 ± 7.72	16.20 ± 7.22	F(6,265) = 5.27, *P* < .001, η^2^ = 0.107
DLD social score‡	9.77 ± 6.76	7.86 ± 6.90	10.28 ± 7.48	11.24 ± 7.46	13.52 ± 10.04	17.91 ± 12.05	22.36 ± 11.50	F(6,259) = 11.16, *P* < .001, η^2^ = 0.205

NOTE. Ages and scores given are mean ± standard deviation. Group comparisons included premorbid ID severity and multimorbidity as covariates.

Abbreviations: ABS, Adaptive Behavior Scale; BRIEF-A, Behavior Rating Inventory of Executive Function–Adult version; DLD, Dementia Questionnaire for People with Learning Disabilities; ID, intellectual disability; IED, intra-/extra-dimensional set shift; KBIT-2, Kaufmann Brief Intelligence Test 2; NEPSY-II, Developmental NEuroPSYchological Assessment-II; OMQ, Observer Memory Questionnaire; PAL, paired associates learning; SRT, simple reaction time.

∗ Assessed via carer report based on everyday functional descriptions.

† Percentages calculated excluding missing values.

‡ Higher scores represent poorer ability.

Comparing the older age groups with those aged 16-30 years (Table 3 and Supplementary Table 2), the earliest changes in performance were seen for the PAL first trial memory score and SRT latency SD, with significantly poorer performance starting in adults aged 41-45 years (*P* = .002 and *P* = .001, respectively). Performance for the majority of other outcomes became significantly poorer for adults aged 46–50 years, with the exception of several informant-rated adaptive ability scores and BRIEF-A scores. By 51–55 years, all measures except BRIEF-A scores showed significantly poorer performance.

**Table 3:** Summary heat map demonstrating age groups where scores are significantly poorer than those for adults aged 16-30 years

Domain	Outcomes	36–40 years	41–45 years	46–50 years	51–55 years	56–60 years
IQ	KBIT-2 verbal raw score			**	***	***
	KBIT-2 nonverbal raw score			***	***	***
Memory	PAL first trial memory score		**	***	***	***
	PAL stages completed		*	***	***	***
	Orientation			**	***	***
	Object memory immediate recall			**	***	***
	Object memory delayed recall			**	***	***
	DLD cognitive score			***	***	***
	OMQ total score			**	***	***
Executive function	IED stage 1 errors		*	***	***	***
	IED stages completed			**	***	***
	Verbal fluency			***	***	***
	Tower of London			***	***	***
	BRIEF-A total score					
	BRIEF-A Behavioral Regulation Index					
	BRIEF-A Metacognition Index					
Attention	SRT total correct			***	***	***
	SRT mean latency	*	*	***	***	***
	SRT latency standard deviation		**	***	***	***
Motor	Finger-nose pointing		*	***	***	***
	NEPSY-II train and car			***	***	***
	NEPSY-II car and motorbike			***	***	***
Adaptive	Short ABS total score			**	***	***
	Short ABS personal self-sufficiency			*	***	***
	Short ABS community self-sufficiency			**	**	***
	Short ABS personal-social responsibility				**	**
	DLD social score			*	***	***

NOTE. ^∗^*P* <.05, **P < .01, and ***P < .001. Results are obtained from post hoc comparisons of ANCOVA results comparing age groups and including premorbid ID severity and multimorbidity as covariates.

Abbreviations: ABS, Adaptive Behavior Scale; BRIEF-A, Behavior Rating Inventory of Executive Function–Adult version; DLD, Dementia Questionnaire for People with Learning Disabilities; ID, intellectural disability; IED, intra-/extra-dimensional set shift; KBIT-2, Kaufmann Brief Intelligence Test 2; NEPSY-II, Developmental NEuroPSYchological Assessment-II; OMQ, Observer Memory Questionnaire; PAL, paired associates learning; SRT, simple reaction time.

### 3.2 Markers associated with clinical stage of AD

Ages and scores for each outcome measure for 170 adults aged 36+ years split into preclinical, prodromal, and clinical dementia groups can be seen in Table 4, with results of group comparisons in Table 4 and Table 5 and an example of group changes in Fig. 1. Participants with no CAMDEX-DS data (n = 8) or when decline was potentially due to another cause such as depression (n = 10) were excluded from analyses.

**Table 4:** Ages and scores for each outcome for adults aged 36+ years split into preclinical, prodromal, and clinical dementia groups, with results of group comparisons for preclinical and prodromal dementia, and prodromal and clinical dementia

Demographics and outcomes	Preclinical dementia	Prodromal dementia	Clinical dementia	Preclinical dementia versus prodromal dementia	Prodromal dementia versus clinical dementia
Total number	68	46	56	N/A	N/A
Number who failed hearing or vision test	4 (5.9%)	4 (8.7%)	6 (10.7%)	N/A	N/A
Age (years)	45.93 ± 6.13	51.28 ± 7.16	54.45 ± 7.00	t(112) = 4.27, *P* < .001	t(100) = 2.25, *P* = .027
KBIT-2 verbal raw score	30.61 ± 18.03	21.81 ± 16.27	9.40 ± 11.81	F(1,101) = 1.20, *P* = .276, η^2^ = 0.012	F(1,87) = 16.01, *P* < .001, η^2^ = 0.155
KBIT-2 nonverbal raw score	13.16 ± 6.72	9.15 ± 6.30	4.40 ± 6.00	F(1,97) = 1.86, *P* = .176, η^2^ = 0.019	F(1,83) = 11.86, *P* = .001, η^2^ = 0.125
PAL first trial memory score	8.58 ± 6.26	2.71 ± 3.15	0.64 ± 1.79	F(1,90) = 13.90, *P* < .001, η^2^ = 0.134	F(1,78) = 9.99, *P* = .002, η^2^ = 0.114
PAL stages completed	5.32 ± 2.96	3.00 ± 2.40	0.96 ± 1.73	F(1,90) = 7.15, *P* = .009, η^2^ = 0.074	F(1,78) = 15.22, *P* < .001, η^2^ = 0.163
Orientation	8.98 ± 4.04	6.15 ± 4.31	2.78 ± 3.84	F(1,96) = 4.64, *P* = .034, η^2^ = 0.046	F(1,84) = 14.30, *P* < .001, η^2^ = 0.145
Object memory immediate recall	8.92 ± 4.05	6.64 ± 4.12	2.24 ± 3.64	F(1,94) = 0.45, *P* = .505, η^2^ = 0.005	F(1,79) = 22.94, *P* < .001, η^2^ = 0.225
Object memory delayed recall	4.93 ± 2.29	3.82 ± 2.33	1.31 ± 2.03	F(1,94) = 0.09, *P* = .763, η^2^ = 0.001	F(1,79) = 23.44, *P* < .001, η^2^ = 0.229
DLD cognitive score∗	8.73 ± 10.04	16.47 ± 10.20	28.31 ± 10.65	F(1,92) = 6.55, *P* = .012, η^2^ = 0.067	F(1,82) = 25.87, *P* < .001, η^2^ = 0.240
OMQ total score∗	73.84 ± 17.21	92.97 ± 16.60	117.74 ± 12.73	F(1,94) = 25.00, *P* < .001, η^2^ = 0.210	F(1,67) = 44.52, *P* < .001, η^2^ = 0.399
IED stage 1 errors∗	6.81 ± 10.04	12.22 ± 11.05	20.72 ± 9.60	F(1,89) = 2.75, *P* = .101, η^2^ = 0.030	F(1,79) = 10.36, *P* = .002, η^2^ = 0.116
IED stages completed	5.75 ± 3.24	3.84 ± 3.52	1.26 ± 2.61	F(1,89) = 1.96, *P* = .165, η^2^ = 0.022	F(1,79) = 12.02, *P* = .001, η^2^ = 0.132
Verbal fluency	9.38 ± 6.40	4.75 ± 4.35	2.54 ± 3.96	F(1,96) = 6.95, *P* = .010, η^2^ = 0.068	F(1,85) = 4.42, *P* = .039, η^2^ = 0.049
Tower of London	6.72 ± 3.39	3.78 ± 3.51	1.42 ± 2.92	F(1,97) = 9.33, *P* = .003, η^2^ = 0.088	F(1,84) = 10.36, *P* = .002, η^2^ = 0.110
BRIEF-A total score∗	116.16 ± 24.44	129.64 ± 20.78	148.00 ± 32.57	F(1,84) = 6.82, *P* = .011, η^2^ = 0.075	F(1,61) = 7.14, *P* = .010, η^2^ = 0.105
BRIEF-A Behavioral Regulation Index∗	51.06 ± 11.94	52.12 ± 9.96	57.42 ± 14.74	F(1,98) = 0.19, *P* = .661, η^2^ = 0.002	F(1,72) = 2.81, *P* = .098, η^2^ = 0.038
BRIEF-A Metacognition Index∗	66.72 ± 14.80	77.58 ± 13.11	89.85 ± 20.11	F(1,85) = 12.99, *P* = .001, η^2^ = 0.133	F(1,61) = 9.08, *P* = .004, η^2^ = 0.130
SRT total correct	83.11 ± 28.56	66.22 ± 31.37	34.29 ± 31.03	F(1,87) = 2.07, *P* = .154, η^2^ = 0.023	F(1,77) = 17.09, *P* < .001, η^2^ = 0.182
SRT mean latency∗	1015.60 ± 669.55	1432.80 ± 662.77	2082.42 ± 636.40	F(1,87) = 2.46, *P* = .121, η^2^ = 0.027	F(1,77) = 16.41, *P* < .001, η^2^ = 0.176
SRT latency standard deviation∗	438.94 ± 267.64	674.31 ± 211.57	839.48 ± 216.29	F(1,87) = 12.36, *P* = .001, η^2^ = 0.124	F(1,77) = 8.37, *P* = .005, η^2^ = 0.098
Finger-nose pointing	8.25 ± 5.27	6.32 ± 5.21	2.60 ± 4.33	F(1,95) = 0.13, *P* = .725, η^2^ = 0.001	F(1,83) = 10.97, *P* = .001, η^2^ = 0.117
NEPSY-II train and car	13.57 ± 6.96	10.18 ± 6.57	3.94 ± 6.68	F(1,93) = 0.25, *P* = .621, η^2^ = 0.003	F(1,82) = 14.46, *P* < .001, η^2^ = 0.150
NEPSY-II car and motorbike	12.68 ± 9.88	6.27 ± 5.60	2.59 ± 5.82	F(1,92) = 3.98, *P* = .049, η^2^ = 0.041	F(1,81) = 6.37, *P* = .014, η^2^ = 0.073
Short ABS total score	80.39 ± 20.96	63.47 ± 21.86	40.82 ± 24.28	F(1,97) = 5.52, *P* = .021, η^2^ = 0.054	F(1,84) = 21.22, *P* < .001, η^2^ = 0.202
Short ABS personal self-sufficiency	28.40 ± 4.84	25.10 ± 6.99	16.33 ± 9.65	F(1,100) = 2.02, *P* = .159, η^2^ = 0.020	F(1,86) = 18.01, *P* < .001, η^2^ = 0.173
Short ABS community self-sufficiency	28.97 ± 11.62	20.24 ± 10.51	11.55 ± 9.16	F(1,99) = 6.38, *P* = .013, η^2^ = 0.061	F(1,86) = 18.47, *P* < .001, η^2^ = 0.177
Short ABS personal-social responsibility	23.02 ± 6.40	18.90 ± 6.44	12.94 ± 7.21	F(1,100) = 3.01, *P* = .086, η^2^ = 0.029	F(1,86) = 19.78, *P* < .001, η^2^ = 0.187
DLD social score∗	9.36 ± 6.31	13.85 ± 8.51	24.76 ± 12.66	F(1,95) = 4.04, *P* = .047, η^2^ = 0.041	F(1,83) = 15.92, *P* < .001, η^2^ = 0.161

NOTE. Ages and scores given are mean ± standard deviation. Group comparisons included age, premorbid ID severity, and multimorbidity as covariates.

Abbreviations: ABS, Adaptive Behavior Scale; BRIEF-A, Behavior Rating Inventory of Executive Function–Adult version; DLD, Dementia Questionnaire for People with Learning Disabilities; ID, intellectual disability; IED, intra-/extra-dimensional set shift; KBIT-2, Kaufmann Brief Intelligence Test 2; NEPSY-II, Developmental NEuroPSYchological Assessment-II; OMQ, Observer Memory Questionnaire; PAL, paired associates learning; SRT, simple reaction time.

∗ Higher scores represent poorer ability.

**Table 5:** Summary heat map demonstrating effect sizes of group differences comparing adults aged 36+ years with preclinical and prodromal dementia, and prodromal and clinical dementia

Domain	Outcomes	Preclinical versus prodromal	Prodromal versus clinical
IQ	KBIT-2 verbal raw score		**
	KBIT-2 nonverbal raw score		*
Memory	PAL first trial memory score	*	*
	PAL stages completed		**
	Orientation		*
	Object memory immediate recall		***
	Object memory delayed recall		***
	DLD cognitive score		***
	OMQ total score	***	***
Executive function	IED stage 1 errors		*
	IED stages completed		*
	Verbal fluency		
	Tower of London		*
	BRIEF-A total score		*
	BRIEF-A Behavioral Regulation Index		
	BRIEF-A Metacognition Index	*	*
Attention	SRT total correct		**
	SRT mean latency		**
	SRT latency standard deviation	*	
Motor	Finger-nose pointing		*
	NEPSY-II train and car		**
	NEPSY-II car and motorbike		
Adaptive	Short ABS total score		***
	Short ABS personal self-sufficiency		**
	Short ABS community self-sufficiency		**
	Short ABS personal-social responsibility		**
	DLD social score		**

NOTE. ^∗^η^2^ > 0.10, ^†^η^2^ > 0.15, and ^‡^η^2^ > 0.20. Group comparisons included age, premorbid ID severity, and multimorbidity as covariates.

Abbreviations: ABS, Adaptive Behavior Scale; BRIEF-A, Behavior Rating Inventory of Executive Function–Adult version; DLD, Dementia Questionnaire for People with Learning Disabilities; ID, intellectual disability; IED, intra-/extra-dimensional set shift; KBIT-2, Kaufmann Brief Intelligence Test 2; OMQ, Observer Memory Questionnaire; NEPSY-II, Developmental NEuroPSYchological Assessment-II; PAL, paired associates learning; SRT, simple reaction time.

Adults with prodromal dementia performed significantly poorer than those with preclinical dementia for several memory, executive function, and attention outcomes. The largest effect sizes were found for the PAL first trial memory score, SRT latency SD, OMQ score, and BRIEF-A Metacognition Index, where group accounted for more than 12% of variance in scores for each outcome.

Adults with clinical dementia performed significantly poorer than those with prodromal dementia on all outcomes aside from verbal fluency, BRIEF-A total score and Behavioral Regulation Index, and Developmental NEuroPSYchological Assessment-II car and motorbike score. The largest effect sizes were for memory measures (object memory, OMQ, and DLD cognitive scores) and short ABS total score, where group accounted for more than 20% of variance in scores for each outcome.

### 3.3 Sensitivity of cognitive markers to *APOE* genotype

Based on preceding analyses, we identified PAL first trial memory score and SRT latency SD as outcomes most sensitive to early AD progression. We compared performance for these outcomes between adults aged 36+ years with genotype *APOE* ε3/ε3 and *APOE* ε3/ε4. Performance for both outcomes was significantly poorer for adults with genotype *APOE* ε3/ε4, with genotype accounting for approximately 8% of variance in scores (PAL: *APOE* ε3/ε3 M = 5.12, SD = 5.42, n = 84, *APOE* ε3/ε4 M = 2.26, SD = 5.18, n = 35, *F*(1,114) = 9.08, *P* = .003, η^2^ = 0.074; SRT: *APOE* ε3/ε3 M = 579.42, SD = 295.63, n = 87, *APOE* ε3/ε4 M = 739.49, SD = 290.28, n = 32, *F*(1,114) = 9.85, *P* = .002, η^2^ = 0.080).

### 3.4 Sample sizes for RCTs using cognitive markers

Using PAL first trial memory score as a primary outcome measure for an RCT to delay early cognitive decline with scores for adults aged 36–40 years without clinical dementia and for those aged 41-45 years (M = 10.63, SD = 6.72, n = 19; and M = 5.90, SD = 5.97, n = 21, respectively; pooled SD = 6.69), an RCT to delay decline in those aged 36–40 years would need 43 individuals per group to detect a significant treatment effect with 90% power. Using a more cautious group difference of 2.70, 130 individuals per group would be required.

Based on short ABS total scores showing later significant age-related decline, we used this as a primary outcome measure for an RCT to delay later cognitive decline. Using scores for adults aged 46–50 years without clinical dementia and for those aged 51–55 years (M = 70.87, SD = 23.03, n = 39; and M = 54.64, SD = 27.56, n = 36, respectively; pooled SD = 26.43), an RCT to delay decline in those aged 46–50 years would need 56 individuals per group to detect a significant treatment effect with 90% power. Using a more cautious group difference of 10.41, 136 individuals per group would be required.

## 4 Discussion

We have investigated cross-sectional changes in cognitive abilities associated with AD development in over 300 adults with DS. Memory and attention measures were most sensitive to aging, with significantly poorer performance starting in the early 40s. Similarly, performance for memory and attention outcomes was most sensitive to progression from preclinical to prodromal dementia, whereas performance for memory outcomes was most sensitive to progression from prodromal to clinical dementia. Using outcomes identified as sensitive to AD progression, we estimated possessing an *APOE* ε4 allele accounted for approximately 8% of variance in scores, and modest sample sizes would be sufficient to detect a significant treatment effect to delay cognitive decline in an RCT.

### 4.1 Strengths and limitations

We report results from the largest study of cognitive decline in DS worldwide, using deep cognitive phenotyping to understand progression associated with the development of AD. A major strength of our study is that we have recruited a large, diverse, community sample of adults with DS, with various stages of AD-related decline. Based on prevalence data from Wu and Morris [1], we have recruited approximately one in 85 of all adults with DS in England and Wales, suggesting our sample is likely to be largely representative of adults with DS in the UK. We used a validated, sensitive battery of cognitive tests and took account of potentially confounding vision and hearing difficulties.

We used a cross-sectional approach, with analyses based on the strong rationale of AD neuropathology being increasingly present in adults with DS with aging; nevertheless, our results need to be confirmed with longitudinal assessments as cross-sectional approaches are vulnerable to cohort effects. However, we controlled for age where possible. Furthermore, missing data from cognitive outcomes were not missing at random; adults with clinical dementia were more likely to have scores missing [8]. We reduced this limitation through imputing scores for cognitive tests when adults had not understood test instructions. In addition, while we used a thorough approach to categorize individuals into preclinical and prodromal dementia, the information used relied on retrospective judgments of carers, which may be subject to recall bias. Finally, there are no standardized thresholds of preclinical and prodromal dementia in DS. We adapted concepts from Dubois et al. [25], defining preclinical dementia by the absence of clinical symptoms of AD and the presence of AD biomarker changes. Owing to the presence of amyloid neuropathology in all adults with DS from the mid-30s [2], [4] and the near universal development of dementia [3], we considered all adults with DS aged 36+ years to be in at least a preclinical state, and those showing symptoms of cognitive decline but not reaching the threshold for dementia diagnosis to be in a prodromal state. We were able to show clear cognitive differences between groups defined in this way, providing some validation for our classification.

### 4.2 Cognitive changes associated with dementia in adults with DS

Dementia diagnosis in people with DS may be complicated by variable premorbid ID, in addition to health comorbidities including depression and hypothyroidism, which may present with cognitive changes. Fully understanding the time course of dementia development in DS is essential for better detection and monitoring of cognitive decline, to detect reliable biomarkers for the progression of decline, and for the development of clinical trials. Previous studies have also demonstrated poorer performance associated with aging, cognitive decline, and dementia in adults with DS for various tasks within our battery [23], [28], [29], [30], [31], [32]. Study results have however differed regarding the sequence of cognitive changes. Similar to our results which suggest that changes in memory and attention are the earliest changes associated with dementia development, other studies have reported memory decline as an early change [33], [34], [35], with decline in memory [36], [37] and attention [38] preceding other changes by up to 3 years. In contrast, a recent systematic review suggested executive dysfunction with behavioral and personality changes, caused by early frontal lobe involvement, may precede memory loss [39] (also see [31], [40], [41]). Although these carer-reported changes in behavior and personality are likely to occur relatively early in the course of dementia in DS, it is possible that carers observe such changes earlier than memory changes that may be less obvious in those with more severe IDs and with communication impairments. Differences in test sensitivity may also explain conflicting results.

The earliest changes in both ADAD [6] and sporadic late-onset AD are also memory changes, though a minority of individuals may initially show an atypical cognitive presentation, with behavioral change, language impairment, dyscalculia, or dysexecutive syndrome [6]. The early memory impairments in adults with DS support similar underlying neurological changes in the development of dementia to these other forms of AD and indicate the importance of the population with DS for clinical trials of treatments to prevent or modify AD.

### 4.3 Implications for clinical trials

The recent failure of clinical drug trials directed at amyloid, including anti-amyloid antibodies and BACE inhibition, has been a considerable disappointment given the genetic evidence suggesting reduced BACE cleavage over a lifetime protects against AD [42]. The most prevalent explanation for this failure is that treatments targeting amyloid may only be effective during the extended prodromal phase of AD [43]. The predictable onset of pathology and high incidence of dementia in DS suggest this is a key population for trials in the preclinical or prodromal stage of AD to prevent or delay decline. To date, individuals with DS have been excluded from such trials despite having the most common genetic cause of AD and a considerable burden of disease, in part due to the lack of reliable cognitive outcome data.

Intervention studies to prevent or delay AD-related decline in those with DS depend on determining the optimal age for treatments to be given, outcome measures that are most sensitive to decline, and relevant effect sizes for such outcome measures to determine adequate sample sizes. Our results address these uncertainties, and although longitudinal studies are needed to confirm our findings, they provide valuable data to plan trials to prevent or delay decline. We found that changes in memory and attention, specifically in the CANTAB PAL and SRT tests, are most sensitive to AD progression. These tests have standardized computerized administrations and so would be suitable measures for use in a multicentre clinical trial. RCTs would likely use several outcomes, both assessed directly and via informant report (with the latter particularly important for those with more severe ID [44]), and our results offer a critical first step toward such trials. We estimate modest samples will be sufficient to detect a significant treatment effect to delay cognitive decline, with treatment started in the mid-late 30s to delay early decline (i.e., before the development of amyloid PET pathology [45]) and started in the mid-late 40s to delay later decline (i.e., before the majority of individuals receive a clinical dementia diagnosis [3], [46]). Accounting for the effect of *APOE* ε4 in the trial design may further sharpen effect sizes, as may accounting for amyloid or tau PET pathology [47]. However, these calculations are based on 5-year age bands; a shorter time frame may require larger sample sizes, while interim analyses may help to shorten trial length. Furthermore, trial samples would need to be larger at entry to account for dropout. Nevertheless, our data and previous studies suggest that even when accounting for this, the number of individuals required for a preventative RCT would be feasible to recruit [48], [49], and such trials would likely also be informative for studies of ADAD and sporadic late-onset AD.

**Research in Context**

1. Systematic review: A literature search of PubMed identified 39 studies investigating cognitive changes associated with Alzheimer's disease and aging in Down syndrome. Most studies were small with mixed results, and few investigated prodromal changes. Some identified early changes in memory, and others suggested that changes in executive function and associated behavior occur first.
2. Interpretation: In the largest cross-sectional cognitive phenotyping study to date of 312 adults with Down syndrome, we found that tests of memory and attention were most sensitive to decline from the preclinical to prodromal dementia state. We used our results to identify primary outcomes for randomized controlled trials of treatments to delay cognitive decline and to estimate sample sizes needed for randomized controlled trials to detect significant treatment effects.
3. Future directions: Given the high burden of dementia in Down syndrome, the development of randomized controlled trials to delay cognitive decline is essential, with our results offering a crucial first step toward such trials.
