# Supplementary material for: Cognitive markers of preclinical and prodromal Alzheimer's disease in Down syndrome
Source: Alzheimers Dement. 2019 Feb;15(2):245–57. doi: 10.1016/j.jalz.2018.08.009 (PMC6374283; doi:10.1016/j.jalz.2018.08.009)
Supplement: Supplementary Tables 1 and 2 [file mmc1.doc]

| **Outcomes** | **16-30 years** | **31-35 years** | **36-40 years** | **41-45 years** | **46-50 years** | **51-55 years** | **56-60 years** |
| --- | --- | --- | --- | --- | --- | --- | --- |
| **Total number excluding those who failed the vision or hearing test** | 92 | 29 | 25 | 23 | 50 | 36 | 27 |
| **KBIT-2 verbal raw score** | 1/92 | 0/29 | 2/25 | 1/23 | 9/50 | 5/36 | 11/27 |
| **KBIT-2 non-verbal raw score** | 0/92 | 0/29 | 0/23 | 1/22 | 6/47 | 5/35 | 11/26 |
| **PAL first trial memory score** | 1/82 | 0/27 | 3/22 | 1/21 | 8/41 | 12/31 | 12/25 |
| **PAL stages completed** | 1/82 | 0/27 | 3/22 | 1/21 | 8/41 | 12/31 | 12/25 |
| **Orientation** | 1/85 | 0/29 | 1/23 | 1/21 | 9/47 | 10/35 | 12/26 |
| **Object memory immediate recall** | 1/81 | 0/29 | 2/22 | 2/20 | 8/46 | 11/34 | 12/25 |
| **Object memory delayed recall** | 1/81 | 0/29 | 2/22 | 2/20 | 8/46 | 11/34 | 12/25 |
| **IED stage 1 errors** | 1/84 | 0/26 | 3/22 | 2/20 | 9/42 | 12/33 | 12/24 |
| **IED stages completed** | 1/84 | 0/26 | 3/22 | 2/20 | 9/42 | 12/33 | 12/24 |
| **Verbal fluency** | 1/86 | 0/29 | 1/23 | 1/21 | 8/48 | 11/35 | 12/27 |
| **Tower of London** | 2/87 | 1/28 | 1/22 | 2/22 | 11/46 | 14/36 | 15/26 |
| **SRT total correct** | 1/82 | 0/24 | 3/23 | 1/19 | 9/41 | 13/31 | 13/21 |
| **SRT mean latency** | 1/82 | 0/24 | 3/23 | 1/19 | 9/41 | 13/31 | 13/21 |
| **SRT latency standard deviation** | 1/82 | 0/24 | 3/23 | 1/19 | 9/41 | 13/31 | 13/21 |
| **Finger-nose pointing** | 1/88 | 0/29 | 1/24 | 2/21 | 8/46 | 12/35 | 12/26 |
| **NEPSY-II train and car** | 0/89 | 0/29 | 1/22 | 2/21 | 9/47 | 11/34 | 14/26 |
| **NEPSY-II car and motorbike** | 0/89 | 0/29 | 1/22 | 2/21 | 9/47 | 12/34 | 14/26 |

Supplementary Table 1. The number of individuals with imputed scores and total number included in analysis for each age group.

| **Outcomes** | **16-30 years vs 31-35 years** | **16-30 years vs 36-40 years** | **16-30 years vs 41-45 years** | **16-30 years vs 46-50 years** | **16-30 years vs 51-55 years** | **16-30 years vs 56-60 years** |
| --- | --- | --- | --- | --- | --- | --- |
| **KBIT-2 verbal raw score** | 1·000 | 1·000 | 1·000 | 0·001 | <0·001 | <0·001 |
| **KBIT-2 non-verbal raw score** | 1·000 | 1·000 | 0·134 | <0·001 | <0·001 | <0·001 |
| **PAL first trial memory score** | 1·000 | 1·000 | 0·002 | <0·001 | <0·001 | <0·001 |
| **PAL stages completed** | 1·000 | 0·764 | 0·034 | <0·001 | <0·001 | <0·001 |
| **Orientation** | 1·000 | 1·000 | 0·699 | 0·001 | <0·001 | <0·001 |
| **Object memory immediate recall** | 1·000 | 0·533 | 0·511 | 0·001 | <0·001 | <0·001 |
| **Object memory delayed recall** | 1·000 | 0·471 | 0·091 | 0·003 | <0·001 | <0·001 |
| **DLD cognitive score** | 1·000 | 1·000 | 0·349 | <0·001 | <0·001 | <0·001 |
| **OMQ total score** | 1·000 | 1·000 | 1·000 | 0·001 | <0·001 | <0·001 |
| **IED stage 1 errors** | 1·000 | 1·000 | 0·032 | <0·001 | <0·001 | <0·001 |
| **IED stages completed** | 1·000 | 1·000 | 0·434 | 0·002 | <0·001 | <0·001 |
| **Verbal fluency** | 1·000 | 1·000 | 0·637 | <0·001 | <0·001 | <0·001 |
| **Tower of London** | 1·000 | 1·000 | 0·824 | <0·001 | <0·001 | <0·001 |
| **BRIEF-A total score** | 1·000 | 1·000 | 1·000 | 1·000 | 1·000 | 1·000 |
| **BRIEF-A behavioural regulation index** | 1·000 | 1·000 | 1·000 | 0·923 | 1·000 | 1·000 |
| **BRIEF-A metacognition index** | 1·000 | 1·000 | 1·000 | 1·000 | 1·000 | 1·000 |
| **SRT total correct** | 1·000 | 0·136 | 1·000 | <0·001 | <0·001 | <0·001 |
| **SRT mean latency** | 1·000 | 0·035 | 0·014 | <0·001 | <0·001 | <0·001 |
| **SRT latency standard deviation** | 1·000 | 0·132 | 0·001 | <0·001 | <0·001 | <0·001 |
| **Finger-nose pointing** | 1·000 | 0·298 | 0·023 | <0·001 | <0·001 | <0·001 |
| **NEPSY-II train and car** | 1·000 | 1·000 | 1·000 | <0·001 | <0·001 | <0·001 |
| **NEPSY-II car and motorbike** | 1·000 | 1·000 | 0·114 | <0·001 | <0·001 | <0·001 |
| **Short ABS total score** | 1·000 | 1·000 | 1·000 | 0·008 | <0·001 | <0·001 |
| **Short ABS personal self-sufficiency** | 1·000 | 1·000 | 1·000 | 0·018 | <0·001 | <0·001 |
| **Short ABS community self-sufficiency** | 1·000 | 1·000 | 1·000 | 0·008 | 0·009 | <0·001 |
| **Short ABS personal-social responsibility** | 1·000 | 1·000 | 1·000 | 0·168 | 0·009 | 0·003 |
| **DLD social score** | 1·000 | 1·000 | 1·000 | 0·022 | <0·001 | <0·001 |

Supplementary Table 2. Pairwise comparisons for mean scores of age groups compared to those of adults aged 16-30 years; values given are *P* values. Group comparisons included pre-morbid ID severity and multi-morbidity as covariates.
